# A mixed-methods study among adolescents and teachers in Bogotá, Colombia: adapting the OurFutures Alcohol Program

**DOI:** 10.1093/heapro/daae152

**Published:** 2024-11-15

**Authors:** Lyra Egan, Laura Ospina-Pinillos, Katrina Elizabeth Champion, Nicola Clare Newton, Paula Valentina Ballen Alonso, Maree Teesson, Lauren Anne Gardner

**Affiliations:** The Matilda Centre for Research in Mental Health and Substance Use, Level 6, Jane Foss Russell Building, The University of Sydney, Sydney, NSW 2006, Australia; Faculty of Medicine, Department of Psychiatry and Mental Health, Pontificia Universidad Javeriana, Av. Alberto Lleras Camargo #40 - 62, Bogotá, Colombia; The Matilda Centre for Research in Mental Health and Substance Use, Level 6, Jane Foss Russell Building, The University of Sydney, Sydney, NSW 2006, Australia; The Matilda Centre for Research in Mental Health and Substance Use, Level 6, Jane Foss Russell Building, The University of Sydney, Sydney, NSW 2006, Australia; Faculty of Medicine, Department of Psychiatry and Mental Health, Pontificia Universidad Javeriana, Av. Alberto Lleras Camargo #40 - 62, Bogotá, Colombia; The Matilda Centre for Research in Mental Health and Substance Use, Level 6, Jane Foss Russell Building, The University of Sydney, Sydney, NSW 2006, Australia; The Matilda Centre for Research in Mental Health and Substance Use, Level 6, Jane Foss Russell Building, The University of Sydney, Sydney, NSW 2006, Australia

**Keywords:** Colombia, prevention, alcohol, eHealth, adolescents, mixed-methods approach, adaptation

## Abstract

Early onset of alcohol consumption among Colombian adolescents highlights the need for effective and accessible preventive interventions. This project aimed to conduct formative work to inform the adaptation of an effective eHealth alcohol use prevention program originally developed in Australia, the *OurFutures Alcohol Module,* to the Bogotá context. Twenty-six adolescents and 10 teachers in Bogotá participated in the study. We used a mixed-methods approach comprising interviews, surveys and semi-structured discussions to evaluate the acceptability of OurFutures. Study materials were translated into Spanish before conducting three 1.5-hour focus groups with adolescents (aged 11–15; *n* = 26), and 1-hour interviews or online surveys with teachers to assess attitudes towards alcohol use and the acceptability of one lesson from the *OurFutures Alcohol Module* in the Bogotá context. Qualitative data were analysed thematically, and descriptive analyses of quantitative data reported percentage agreement for survey questions. Overall, 96% of students and 89% of teachers expressed strong satisfaction with the *OurFutures Alcohol Module* lesson. Most students (96%) liked its storyline and character portrayal, and most teachers (80%) said they would use *OurFutures* with their students. Participants provided feedback for improving program relatability, including adapting scenarios, character names, clothing and language to align with the Bogotá context and resonate with Colombian adolescents. This study marks the first step in informing the adaptation of the *OurFutures Alcohol Module* to the Bogotá context and highlights key considerations for cultural adaptations of other substance use prevention interventions. This research underscores the importance of place-based end-user involvement in co-designing adolescent prevention interventions.

**Contribution to Health Promotion**

- Colombian national survey data has reported early alcohol use initiation among adolescents, yet standardized alcohol prevention programs are lacking in Colombian schools.
- This study formatively assessed the acceptability of one lesson of the *OurFutures Alcohol Module*, an effective eHealth school-based alcohol use prevention program originally developed in Australia, within the Bogotá context.
- The findings provide evidence that, with appropriate adaptation involving co-design with place-based end users, the *OurFutures Alcohol Module* could be an acceptable and engaging alcohol prevention strategy in the Bogotá context.
- This study offers valuable insights and recommendations for developing culturally tailored preventive interventions.

## INTRODUCTION

Alcohol consumption during adolescence is associated with increased risk of alcohol-related harms and future substance use (Ryan *et al*., 2019; Hamidullah *et al*., 2020; Australian Institute of Health and Welfare, 2021). According to the 2019 Colombian National Survey on the Use of Psychoactive Substances (ENCSPA), 46.3% of young people aged 12 to 17 years had consumed alcohol at least once, with 12.1% over the past 30 days (National Administrative Department of Statistics. DANE, 2020). Alarmingly, the average age of first alcohol consumption among this cohort is 13.9 years. Initiation at 14 years or younger among Colombian youth has been associated with two-and-a-half times the risk of hazardous alcohol use and four times the risk of substance use in adulthood, compared to those who first consume alcohol at 18 years (Pérez Gómez *et al*., 2011). This trend aligns with Australian findings, where earlier initiation is associated with hazardous drinking at a younger age (Gardner *et al*., 2023). According to the recent WHO Global Status Report on Alcohol and Health and Treatment of Substance Use Disorders (World Health Organization, 2024), 23.7% of young people aged 15–19 years in Colombia are current drinkers, which is higher than in neighbouring countries such as Guatemala (17.4%) and Honduras (19.6%). Additionally, 12.4% of young Colombians aged 15–19 years report binge drinking, defined as consuming 60 grams or more of pure alcohol (equivalent to six standard drinks) at least once in the past month. Although the 12.4% rate of binge drinking among young Colombians is lower than in neighbouring countries such as Argentina (28.3%), it remains a significant public health concern (World Health Organization, 2024). Binge drinking among young people is associated with acute consequences such as alcohol poisoning, motor vehicle accidents and violence, as well as long-term consequences including alterations to general brain functioning and substance use dependence (Kuntsche *et al*., 2017; World Health Organization, 2024). Factors that may influence drinking patterns in Colombia include cultural and social normalization of alcohol consumption, positive attitudes towards alcohol, and low-risk perception (Londoño-Pérez and Carrasco-Aravena, 2019). Early intervention and prevention is therefore critical for safeguarding the health of Colombian youth.

School-based alcohol prevention interventions are an effective approach for delivering long-lasting health and economic benefits (Kuklinski *et al*., 2021; Newton *et al*., 2022). Ensuring successful delivery of prevention interventions involves careful consideration of available resources (e.g. personnel, training, and financial costs), and the target population’s needs. Given approximately one in four of the Colombian population lives in rural regions (National Administrative Department of Statistics. DANE, 2022), electronic health (eHealth) interventions (e.g. computer-, web-, mobile-, or telephone-based) may be a viable option as they can be accessed remotely and at little cost to the end user, and provide increased implementation fidelity and student engagement (Newton *et al*., 2017). There is substantial evidence to support the effectiveness of school-based eHealth interventions in reducing alcohol use and intentions to use alcohol (Voogt *et al*., 2013; Hutton *et al*., 2020; Martinez-Montilla *et al*., 2020; Kazemi *et al*., 2021; Newton *et al*., 2022). However, to our knowledge, there are currently no school-based eHealth interventions targeting alcohol use among Colombian youth, nor are there standardized alcohol prevention programs established in Colombian schools.

Developing a sustainable and effective prevention intervention can be expedited by building upon evidence-based programs known to be effective at driving behaviour change. The Australian *OurFutures prevention model* (formerly known as Climate Schools) has been used to develop a suite of web-based substance use prevention programs for school-aged students. The *OurFutures* model was developed by researchers at Australian universities funded by government and philanthropic grants. The development involved collaboration with students, teachers, and health and education experts, aimed at offering programs that overcome barriers to implementing high-fidelity programs to address the critical need for early prevention of substance use (Newton *et al*., 2009; Vogl *et al*., 2012). Based on a harm minimization and social influence approach, the core component of the program utilizes interactive online cartoon storyboards to engage and educate students, with optional teacher-facilitated activities to reinforce the core content. The *OurFutures* programs have been rigorously evaluated across eight large cluster randomized controlled trials in Australia (240 schools, >21 000 students, up to 7-year follow-up), demonstrating effectiveness in preventing the uptake and harmful use of alcohol and other drugs, reducing harms related to alcohol and other drugs, and improving attitudes towards alcohol among Australian adolescents (Newton *et al*., 2020; Slade *et al*., 2020), up to 7 years after intervention delivery (Newton *et al*., 2022). This model offers promise for international adaptation, with adapted versions already developed for multiple countries, including the UK (Newton *et al*., 2014) and Germany (Röhrig *et al*., 2023). However, recognizing that the *OurFutures Alcohol Module* was originally designed for Australian adolescents aged 13–14 years, and the average age of first alcohol consumption in Colombia is 13.9 years, potential adjustments for earlier delivery may be warranted to align with the cultural and developmental context of Colombian adolescents.

Considering the diversity of Colombia’s regions, Bogotá, the nation’s capital and largest city, stands out as an ideal starting point for conducting this formative work. With over 8 million inhabitants from various parts of the country, Bogotá’s diverse population presents an opportunity to encounter a broad spectrum of races and cultures on a smaller scale (National Administrative Department of Statistics. DANE, n.d.). To ensure the acceptability and feasibility of the *OurFutures Alcohol Module* within the Bogotá context, we first require an understanding of the individual, interpersonal and cultural beliefs associated with alcohol consumption among Bogotán youth (Londoño-Pérez and Carrasco-Aravena, 2019) and feedback from young people and teachers on the program in its current form. Collaborating with and including end users in the intervention design process is crucial, as it can influence the development, engagement and outcomes of an intervention (Slattery *et al*., 2020). Therefore, the aim of this study was to seek feedback from Bogotán young people and teachers as a first step towards guiding the adaptation of an effective eHealth school-based prevention program (*OurFutures Alcohol Module*) for the Bogotá context.

## METHODS

### Study design

This study followed a mixed-methods case-study design, collecting data from focus group sessions with students and interviews or online surveys with teachers. This approach was chosen based on established frameworks and methodologies for effective adaptation led by team members N.C.N., M.T. (Newton *et al*., 2014) and L.O.-P. (Ospina-Pinillos *et al*., 2020), facilitating a comprehensive exploration of participant attitudes toward alcohol use, the acceptability of *OurFutures* and identifying areas of improvement to suit the Bogotá context. This study was approved by the University of Sydney Human Research Ethics Committee (HREC 2022/875) and Research and Ethics Committee of Pontificia Universidad Javeriana (HREC 2023/105).

### Participants and recruitment

One school in Bogotá was invited to participate via an invitation letter to the school principal in July 2023. This specific school was chosen due to established professional relationships between the school and the research team and because they had previously expressed interest in participating in research studies. The school is a large public school catering to students attending Preschool, Primary School (grades 1–5), Lower Secondary School (grades 6–9), and Upper Secondary School (grades 10–11). The school primarily serves populations facing socioeconomic disadvantage, including those experiencing economic hardship and migrants. This context is particularly significant as research indicates that socioeconomic disadvantage is associated with increased substance use, including alcohol consumption (Degenhardt *et al*., 2016). Therefore, selecting this school allowed for the provision of evidence-based resources to an underserved population that may have limited access to prevention programs, and the findings may therefore be especially important for populations at higher risk of alcohol use.

Eligible participants were adolescents aged 11–15 years proficient in Spanish for focus groups, and teachers for interviews or online surveys. Students from grades 5–7 were selected by their teachers to participate, and information about the study was disseminated to school staff via the primary contact. Two additional teachers from separate secondary schools in Bogotá were recruited following L.O.-P.’s lecture to Master of School Mental Health students, where the study was promoted to ensure a sufficient number of teachers participated in the study. Adolescents were required to obtain written parental consent and give their assent to participate, and teachers were required to provide active consent. As reimbursement for their time, students received scholarly goods valued at COP$30.000 (approximately AUD$10) and teachers received a COP$50.000 (approximately AUD$17) voucher for their time.

### Procedures

Study materials, including focus group and interview guides, online surveys for adolescents and teachers, and an alcohol factsheet for adolescents, were translated by a native English speaker fluent in Spanish (L.E.). These translations were then reviewed by an official Spanish translator to ensure accuracy, followed by review for linguistic and cultural relevance by the Colombian researchers (L.O.-P. and P.V.B.A.). The *OurFutures Alcohol Module* was translated into Spanish using the in-built translation software, ‘Kanzi’, on the *OurFutures* website. As a precaution for potential internet connectivity issues at the participating school or for teachers, one lesson was also downloaded as a hardcopy PDF version. Subsequently, between August and October 2023, focus groups and interviews were facilitated in Spanish by the research team (L.E., L.O.-P., P.V.B.A.), guided by a semi-structured format, and audio-recorded for transcription. All surveys were administered in Spanish via REDCap.

Three 1.5-hour student focus groups were held at the secondary school in Bogotá. Part one of each session explored participants’ attitudes and perceptions towards alcohol use. Subsequently, participants reviewed one lesson from the *OurFutures Alcohol Module* and provided feedback on the program’s relatability and acceptability within the Bogotá context. Towards the end of the session, participants completed a brief evaluation survey and commented on the acceptability of an age-appropriate debrief alcohol factsheet. Participants could keep the debrief factsheet, which contained factual information about alcohol including its effects and potential risks of alcohol consumption during adolescence, as well as contact information for L.O.-P. should they require further information. L.O.-P. is a registered psychiatrist and was available as part of duty of care protocols to guide participants towards appropriate help if needed. Additionally, participants were provided with contact details for separate support services accessible via email, WhatsApp or over the phone, ensuring they had access to helpful resources.

Teachers reviewed one lesson from the *OurFutures Alcohol Module* prior to participating in the 1-hour Zoom interview or online survey (based on their preference). Both the interview and survey featured similar questions as detailed in Supplementary Appendices pg. 7–32. Part one of the interview or online survey focused on attitudes and perceptions about alcohol consumption among young Colombians and alcohol education in Colombian schools. Subsequently, teachers provided feedback on the relatability and acceptability of the *OurFutures Alcohol Module* lesson within the Bogotá context.

### Materials

The *OurFutures Alcohol Module* is a universal eHealth school-based program, originally developed for Australian adolescents aged approximately 13–14 years. Consisting of six cartoon-based lessons with accompanying teacher-facilitated activities aimed at decreasing alcohol misuse, the module is embedded within the school health curriculum and requires no formal teacher training. A summary of lesson content is provided in Supplementary Appendices (pg. 2).

Due to poor internet speed at the participating school, a lesson from the *OurFutures Alcohol Module* was provided as a hardcopy PDF version in Spanish for all focus group sessions. Most participating teachers evaluated the same hardcopy PDF version for this reason, with the exception of two teachers from different schools who reviewed the lesson via the *OurFutures* website. Focus group participants received a printed one-page alcohol factsheet to review and keep (see Supplementary Appendices pg. 3–4). The focus group and interview guides, and online surveys administered to adolescents and teachers, can be found in Supplementary Appendices (pg. 5–36).

### Analysis

Quantitative data were analysed using R version 4.2.2. Descriptive analyses were performed to report sample characteristics and percentage agreement for each survey item. Qualitative data including transcripts from focus groups and interviews, along with collected open-ended survey data were analysed using reflexive thematic analysis in NVivo by two researchers (L.E. and P.V.B.A.) to construct key themes and insights (Braun and Clarke, 2006; Terry *et al*., 2017; Braun *et al*., 2023). The research team adopted an experiential orientation to the data by coding significant quotes independently and then discussing coding differences to improve reflexivity and develop richer interpretations, before developing and grouping meaning-driven themes. This inductive approach allowed for participant-driven discussion priorities, ensuring a genuine representation of feedback. Data were anonymized to protect confidentiality. L.E. and P.V.B.A. recognized their varying assumptions that may have influenced the analysis. L.E., a native English speaker with Spanish as a second language and substantial experience in Australian research contexts, particularly in chronic disease risk behaviours among young people, may have brought a broader, more external perspective to the research. In contrast, P.V.B.A., a native of Bogotá with extensive research experience in Colombia among diverse population groups and research topics, contributed a localized interpretation likely influenced by their lived experience in Bogotá. While these perspectives enriched the analysis, they also reflect the subjectivity inherent in L.E. and P.V.B.A.’s roles as researchers.

## RESULTS

A total of 26 students from grades 5, 6, and 7 participated in three focus groups. Most adolescents identified as male (59%) and were aged between 11 and 15 years (*M* = 12.7 years; SD = 1.2). A total of 10 teachers participated, with 3 opting for interviews and 7 completing the online survey. Among these teachers, 8 were from the same school as the focus group participants, and 2 were from two other schools in Bogotá. The participants’ teaching experience ranged from 8 to 28 years (*M* = 19.9 years; SD = 8.4).

### Part 1: Student and teacher attitudes and perceptions towards alcohol use

Quantitative data from the teacher surveys and interviews revealed that all teachers (10/10; 100%) perceived that drinking alcohol was common among Colombian youth and that it co-occurs with other risk behaviours. More than half (6/9; 67%) perceived alcohol consumption patterns as similar across different geographical locations (cities vs rural areas); however, only 40% (4/10) perceived alcohol consumption patterns as similar across different socioeconomic status levels. Awareness of evidence-based alcohol prevention resources for young Colombians was limited (1/10; 10%); however, most teachers (8/10; 80%) reported a high likelihood of using such programs if available. Refer to Supplementary Appendix Table 3 pg. 37 for further details.

Table 1 summarizes the four key themes that were generated from the student focus group discussions and teacher interviews/surveys, revealing shared perspectives of the cultural significance of alcohol. Detailed insights are available in Supplementary Appendices (pg. 38–40). Socioeconomic status was identified as having a greater influence than geographical differences on the initiation age and type of alcohol consumed. Despite awareness of health impacts and risks, one teacher emphasized the low-risk perception of alcohol in Colombian society, especially among minors.

**Table 1:** Summary of key themes related to student and teacher attitudes and perceptions towards alcohol consumption among young Colombians

Theme	Description	Example quote
Cultural significance of alcohol	Alcohol plays a prominent role in Colombian culture, often present in celebrations and gatherings. Early exposure is common, and may be viewed by parents as promoting responsible consumption.	‘*Unfortunately, alcohol is part of Colombian culture, it’s seen in any kind of gathering, … it’s considered normal for children and teenagers to consume alcoholic beverages at family gatherings, with some saying it’s better for them to get used to it’.* (Teacher 6)
Developmental stages and initiations	Alcohol consumption among young people is perceived as common. Initiation often occurs in family settings, with minimal geographical variations. Urban areas offer diverse alcoholic beverages, while rural areas have traditional drinks. Socioeconomic status influences onset age and type of alcohol consumed.	‘*Alcohol consumption among young Colombians normally first occurs in family spaces, … [rather than] differences in consumption by geographical locations, … what changes in the socioeconomic strata is the type of liquor consumed, well children from low socioeconomic strata consume more beer or low-priced liquor, while those in higher socioeconomic strata consume higher quality and priced liquor’.* (Teacher 3)
Alcohol consumption influences and practices	Student-perceived motivations for alcohol consumption included depression, seeking independence, rebellion and curiosity. Social modelling—witnessing adults and peer consume alcohol—influences experimentation with alcohol. Students and teachers discussed commonality of combining alcohol with substances including cocaine, cannabis and cigarettes.	‘*… you also see several young people, and it seems that the others inspire them to try that, and well, they seem to like it, … and they keep trying it’.* (Grade 6 focus group)
Health awareness and risk perception	Students perceived immediate risks when young people consume alcohol including violence. Grades 6 and 7 extend concerns to long-term dangers, including cancer. However, some teachers discussed a low-risk perception in Colombian society, especially among minors, attributed to family influence.	‘*… people’s risk perception scales regarding how serious consuming alcohol is, it is inversely proportional. Among all drugs, alcohol has the lowest risk perception, around 10% or a little less. This high frequency of consumption, especially among minors, is often linked to family influence*’. (Teacher 1)

### Part 2: Relatability and acceptability of *OurFutures* within the Bogotá context

Student and teacher data from focus groups and interviews/surveys provided in-depth insights on the acceptability of *OurFutures* within the Bogotá context, including its potential integration into alcohol prevention curriculum in Colombia. This information is described in the following six themes.

(i) *The unique landscape of alcohol prevention in Bogotá schools*

Most teachers reported being unaware of evidence-based alcohol prevention courses for young people in Colombia. Although training based on evidence offered by external organizations such as the Health Department was mentioned, concerns were also expressed about existing programs funded by the alcohol industry and potential conflicts of interest.

‘*Based on evidence, I only know about this one. I also know about training offered by the Pan American Health Organization in Colombia, along with the Health Department of Bogotá.*’ (Teacher 1)

Alcohol education approaches varied among teachers, with no standardized alcohol prevention curriculum in Colombian schools. Some teachers described delivering it based on their judgement, using resources they found online, or involving external psychologists. Conversely, others outlined a comprehensive curriculum starting in fifth grade, covering various dimensions related to alcohol.

‘*Schools provide information for prevention purposes, but the program depends on each institution.*’ (Teacher 8)

‘*…two psychologists from the … Health Department come. They conduct group activities focused on the theme of prevention, … as a whole group intervention. The intervention involves dynamics, games, and discussions about the topic. In cases of specific instances of consumption, we refer them to [the hospital] for further assistance …*’ (Teacher 2)

‘*… In our school, we start in the fifth grade, focusing on identifying protective factors, … We begin by talking about alcohol, touching on the risks at a physical and cerebral level … In the sixth grade, we delve a bit more into the social dimension, in the seventh grade, into the biological dimension, in the eighth grade, into the dimension of human relationships, and in the ninth grade, we integrate it more.*’ (Teacher 1)

Teachers expressed readiness to adopt evidence-based alcohol prevention programs with their students if they were relatable, available, engaging and effectively addressed negative effects of early alcohol consumption.

Turning to the acceptability of the *OurFutures Alcohol Module,* the results from both student and teacher questionnaire data indicated it was generally received positively, with most students (25/26; 96%) and teachers (8/9; 89%) rating the lesson they reviewed as good or very good. Additionally, students reported a strong liking for the storyline (25/26; 96%) and characters (25/26; 96%). Generally, teachers agreed (9/10; 90%) that the educational content within the *OurFutures Alcohol Module* is appropriate for Colombian youth, and that they would use this program with their students (8/10; 80%). Further details on student and teacher acceptability data can be found in Supplementary Appendices (pg. 41).

Analysis of qualitative data (focus group and interview discussions, and open-text responses) supported findings from the questionnaires. Students generally rated the *OurFutures Alcohol Module* lesson highly stating they liked the story, and teachers generally endorsed the program, citing its preventive approach and potential to destigmatize discussions on alcohol.

‘*I would use the proposed program as it allows us to approach the subject in a preventive way and would eliminate the taboo that exists to talk to students about the topic of alcohol consumption.*’ (Teacher 3)

The positive feedback, however, was accompanied with recommendations for improvement, particularly in terms of cultural contextualization (summarized in Table 2).

**Table 2:** Key recommendations from teachers and students to integrate and improve the *OurFutures Alcohol Module* for the Bogotá context

Theme	Recommendations
(i) The unique landscape of alcohol prevention in Bogotá schools	The program must be relatable to young Colombians, engaging and effectively address the negative impacts of early alcohol consumption to encourage teachers to use the program with their students.
(ii) The language needs to be contextualized	Incorporate familiar and culturally relevant language. Avoid overly technical or refined language. Consider integrating jargon and using everyday language used by young Colombians.
(iii) Aligning alcohol representation in *OurFutures* with Colombian context	Adjust the portrayal of alcoholic beverages to better resonate with Colombian culture. Substitute instances of characters drinking vodka with locally familiar drinks like ‘Aguardiente’ and common beer brands.
(iv) Narrative elements and character diversity	Enhance character representation and story elements to align with the Colombian context. Address concerns about the perceived affluence of existing characters. Modify scenes to include diverse and realistic characters, avoiding beauty stereotypes and unrealistic scenarios.
(v) Educational content	Ensure the content’s appropriateness for different age groups from different schools, as some situations may not be suitable for all students.
(vi) Teachers appreciate the flexibility in delivery but call for improvements	Provide additional activities. Ensure platform efficiency and consider the inclusion of follow-up sessions, ongoing evaluation and continuous support and feedback for students.

(ii) *The language needs to be contextualized*

Teachers and students emphasized the importance of using familiar and culturally relevant language in *OurFutures*. While some teachers perceived the existing language as relatable, others felt it was occasionally too technical. Suggestions included incorporating jargon and changing alcohol-related terms to align with everyday language used by young Colombians.

‘*It is necessary to recognise and use vocabulary that is familiar to young Colombians.*’ (Teacher 3)

‘*…here in Colombia, we say “trago,” [equivalent to booze in English] “chorro,” “aguardiente,” nothing too refined or delicate when talking about alcohol … and I see that in the cartoons, there was a language that was either too technical or too refined…*’ (Teacher 2)

‘*It would be very important to have the Colombian accent, the physical appearance of the characters, and their names, also the fashion*…’ (Grade 7 focus group)

Students proposed culturally relatable alternatives for character names, including ‘Sebastián’ for Mike, ‘Valentina’ for Jane, ‘Tomás’ for Tom, ‘Sara’ for Claire, and ‘David’ for Dave. Moreover, they recommended integrating colloquial or Bogotán expressions to substitute words that may not resonate well with Colombian audiences. For instance, they identified the term ‘guay’ (‘cool’ in English) as characteristic of Spanish from Spain, suggesting more typical Colombian expressions to replace it, including ‘chévere’.

(iii) *Aligning alcohol representation in OurFutures with the Bogotá context*

Students recommended aligning the depiction of alcoholic beverages in *OurFutures* with Colombian culture. Suggestions included swapping instances of characters drinking vodka with locally familiar drinks such as ‘Aguardiente’ (brandy in English), premixed drinks, and common beer brands. Only grade 5 students discussed a preference for mixing beer with sweets, a trend they observed on TikTok.

*‘There wouldn’t be vodka, rather aguardiente…’* (Grade 7 focus group)

(iv) *Narrative elements and character diversity*

Students and teachers expressed concerns regarding the misalignment of character representation and storyline elements within the Bogotá context. Despite appreciating some characters, students desired more characters and criticized the perceived affluence of existing ones. Recommendations included changes to clothing, hairstyles and broader diversity in appearances to better represent people of their age.

‘*They were very posh, I would change that.*’ (Grade 5 focus group)

Teachers echoed the need for more realistic and diverse characters, expressing concerns about beauty stereotypes and relationship portrayals.

‘*There were some little things that bothered me, I feel that there are some beauty stereotypes…the only one with a boyfriend in the group is the slim cute girl*.’ (Teacher 1)

Both students and teachers suggested modifying beach scenes (more aligned with Australian contexts) to park environments to better resonate with youth in Bogotá. Students also critiqued a scene involving characters riding a shopping trolley as unrealistic, suggesting alternatives such as riding bikes. Teachers proposed including locations where young people consume alcohol such as family gatherings to better reflect social dynamics in Colombia. While some scenarios were praised by teachers, with one stating they appreciated *OurFutures’* effort in presenting realistic situations, others raised concerns, particularly regarding the portrayal of absent parents.

‘*…it somewhat contradicts what parents might expect. I think, while the situation, for example, about girls drinking, one’s mother being absent, the context of absent parents—parents are absent, but not absent like that…*’ (Teacher 1)

(v) *Educational content*

Teachers generally appreciated the clarity and appropriateness of the content, and commended its diverse representation of authority figures extending beyond the police. This broad representation was perceived as an effective strategy to raise awareness about decision-making and social influence. However, one teacher perceived a lack of religious or spiritual elements, and others expressed concerns about the advanced content, deeming it inappropriate for certain age groups.

‘*Yes, the educational content, because it’s a matter of raising awareness about decision-making … I found it interesting that they didn’t only portray the police as [the only] authority figure … authority figures are also at home, parents, guardians, here at school, teachers, counsellors, coordinators … I consider that part of the pedagogical sense is very well framed*.’ (Teacher 2)

‘*It is very advanced for the age of the children.*’ (Teacher 9)

‘*…I’m also a homeroom teacher for seventh grade, ranging from 13 to 14 years old; it wouldn’t work at all. The content is a bit advanced, and in terms of the situation, showing them that might open windows they’re not ready for yet. They wouldn’t understand the situations very well.*’ (Teacher 1)

(vi) *Teachers appreciate the flexibility in delivery but call for improvements*

Teachers expressed diverse perspectives on the *OurFutures* format. Some valued the 20-min cartoons as the core component and flexibility in completing optional activities, deeming this format suitable for class schedules, especially when time constraints are a concern. Conversely, some called for additional activities, whereas others found the format lengthy and wanted more dynamic and engaging elements. The two teachers who reviewed *OurFutures* via the website said the platform was slow.

‘*…it was cool that the central part was 20 minutes, I think that’s what you can take advantage of in a class. In my classes, they are 50 minutes long, and in the end, between discipline, behaviour issues, what ends up being used in a class is about 20 minutes, … doing more activities is a bit ambitious …*’ (Teacher 1)

Other recommendations from teachers included incorporating follow-up sessions, continuing evaluation for enhanced learning outcomes, and providing ongoing support and feedback.

*‘Follow-up and not abandoning the program.’* (Teacher 4)

*‘Feedback and ongoing evaluation.’* (Teacher 5)

## DISCUSSION

This study formatively assessed the acceptability of one lesson from the *OurFutures Alcohol Module* in the Bogotá context, as well as participant attitudes and perspectives underpinning alcohol consumption among young Colombians to better understand how to guide the adaptation of the program. By gathering insights from Colombian young people and teachers, this study represents the initial step towards informing the adaptation of an effective eHealth school-based program for the Bogotá context. Feedback from 26 students in 3 focus groups and 10 teachers through surveys (7 teachers) and interviews (3 teachers) highlighted the significant impact of cultural and social factors on attitudes toward alcohol among young Colombians. It underscored the importance of programs such as the *OurFutures Alcohol Module* in their context. While overall participants found the *OurFutures Alcohol Module* lesson acceptable and provided positive feedback about the program, several recommendations were made to enhance its cultural relevance. This emphasizes the need for refinements based on participant feedback to ensure optimal effectiveness and resonance within the Bogotá context.

A prevalent theme was the cultural significance of alcohol in Colombian society, with participants discussing its ubiquitous presence in celebrations and gatherings, synonymous with social enjoyment. Participants collectively acknowledged these events as early and normalized exposures for young Colombians. Thus, the consensus among participants on the commonality of alcohol consumption among young Colombians was unsurprising, and also aligned with the 2019 ENCSPA Colombian National Survey findings (National Administrative Department of Statistics. DANE, 2020). Teachers also perceived that geographical and socioeconomic factors influence adolescents’ alcohol preferences, however, emphasized the added impact of socioeconomic status on alcohol initiation among young Colombians. This underscores the importance of universal alcohol prevention interventions portraying variations in alcoholic beverages for relevance in both urban and rural areas, and across socioeconomic status levels. Furthermore, offering earlier program delivery is crucial, particularly due to the earlier age of first alcohol use in Colombia compared to Australia (National Administrative Department of Statistics. DANE, 2020).

The *OurFutures Alcohol Module* is well-suited to address these issues, employing a harm minimization and social influence approach, coupled with attributes associated with positive prevention outcomes (UNODC/WHO, 2018; Ogden and Hagen, 2019). This approach involves providing evidence-based information about alcohol use and associated harms, normative education to correct misperceptions, and resistance skills training. As students cited various motivations for alcohol consumption, ranging from curiosity, seeking independence and social modelling, where young people imitate the alcohol-related behaviours observed in adults and peers [which is consistent with Yuen *et al.* (2020) findings on peer influence in alcohol use], *OurFutures*’ focus on social influence aligns with the experiences articulated by students. Furthermore, *OurFutures* aligns with the 2019 Colombian Comprehensive Policy for Psychoactive Substance Use, which advocates for capacity building within educational communities, strengthening coping skills to resist social pressures related to substance use and supporting the implementation of evidence-based harm reduction strategies (Ministry of Health and Social Protection, 2019).

Teachers discussed the unique landscape of alcohol prevention in Colombian schools, noting a lack of standardized alcohol prevention curricula with varying approaches among them for implementing such education, and limited teacher awareness of evidence-based resources (10%). Most teachers (80%) expressed a high likelihood of using a tailored digital alcohol education program, contingent on relatability, availability and engagement. Together, these observations highlight the imperative of tailoring the *OurFutures Alcohol Module* to the Bogotá context, particularly as most teachers were unaware of evidence-based programs and some of the only programs teachers referenced were funded by the alcohol industry, which have been reported as problematic for minors (Robaina *et al*., 2020).

Student focus group and teacher interview/survey insights into the relatability and acceptability of *OurFutures* within the Bogotá context, demonstrated that overall, *OurFutures* was rated positively, with 96% of students and 89% of teachers rating the lesson highly. Additionally, 96% of students liked the storyline and characters. Most teachers (90%) perceived the content as reasonable and showed a willingness to deliver the program with their students (80%). Both groups identified key areas for improvement, which the research team will address through a detailed, iterative, co-design process, including language contextualization, adjusting depiction of alcoholic beverages to align with Colombian culture, incorporating diverse and realistic characters, modifying narrative elements, adjusting educational content for different age groups, and refining the program format.

Regarding language contextualization, teachers and students stressed the importance of using familiar, colloquial vocabulary and culturally relevant expressions. This includes adjusting alcohol-related terms to match everyday language used by young Colombians, updating character names with culturally relevant alternatives, and integrating colloquial expressions to replace terms that may not have translated well or reflected vocabulary from Spain rather than Colombia. Given the variation in Spanish lexicon between Colombia and Spain (Ardila, 2020), accurate and culturally appropriate language use is essential. Thus, a meticulous language adaptation process, such as the one outlined by Maríñez-Lora *et al.* (Maríñez-Lora *et al*., 2016) that involves not only forward translation but also back-translation and harmonizing the translation to confirm it reflects the original meaning is warranted.

Both students and teachers highlighted the misalignment of character representation and story elements with the Bogotá context, seeking more realistic and diverse characters, critiquing the perceived affluence of existing ones and suggesting changes to clothing, hairstyles, and broader diversity. As feedback was gathered from public school students, the affluence concern may not apply to students from schools in more affluent areas of Bogotá. Nevertheless, to address these considerations, a collaborative approach involving the co-design of new characters with youth in Bogotá is necessary to ensure authenticity and relevance. Modifying unrealistic scenes was perceived as important by both groups to ensure cultural relevance, with recommendations including shifting from beach to park environments—considering Bogotá’s landlocked nature—and including family gatherings as settings for alcohol consumption among youth in Bogotá, aligning with participants’ views on the cultural normalization of alcohol in such situations. Teachers additionally expressed concerns about beauty stereotypes and relationship depictions, which are crucial to address in the cartoons. Adolescence is a formative period where individuals are developing their identities and self-perceptions (Ogden and Hagen, 2019). Adolescent exposure to unrealistic beauty standards can contribute to body image issues and negatively impact self-esteem and mental health (McBride *et al*., 2019). Diverse portrayals of relationships can influence adolescents’ understanding of healthy relationships, fostering positive social dynamics and interpersonal skills (Vaterlaus *et al*., 2018; Taba *et al*., 2020; West *et al*., 2021). Therefore, it is essential to address these concerns through co-design with youth in Bogotá, so that the content remains engaging and relevant, ultimately promoting adolescents’ overall wellbeing.

Teachers typically appreciated the educational content’s clarity and appropriateness, especially the inclusion of various authority figures beyond police, encompassing parents, teachers and counsellors, which is characteristic of the comprehensive approach adopted in *OurFutures*. Concerns raised by a teacher about the inappropriateness of the advanced content for certain age groups suggest comprehensive content testing with school students from diverse backgrounds to align with students’ developmental stages is required. This ensures relevance without compromising educational integrity, as delivering the program at a later age would not precede the average onset of alcohol consumption in Colombia. Although careful consideration of the appropriateness of the content for younger age groups is warranted. Teacher feedback on the *OurFutures* format varied, with some valuing the 20-min cartoons and flexibility, while others finding it lengthy and desiring more dynamism. As most teachers reviewed the PDF version of the cartoons, it is challenging to gauge whether their opinions would remain consistent when viewing the cartoons on the website. Although the website was not without issue as two teachers expressed concerns about the poor platform speed, emphasizing the need for optimized website performance outside of Australia. Additional recommendations included follow-up sessions, continuous evaluation and ongoing support for enhanced learning outcomes.

### Limitations and strengths

It is crucial to interpret the study findings within the context of certain limitations. Despite Bogotá being the most populous and largest city in Colombia, the study findings may not fully capture the country’s regional diversity. Furthermore, the involvement of only one public school for the focus groups may limit representation of the diverse student population both in Bogotá and across Colombia. However, it was important to collaborate with this school as it served disadvantaged adolescents, offering a unique opportunity to offer essential resources to support the needs of this underserved population. A key strength of *OurFutures* is that it is built on complex models of behaviour change that are embedded within the storylines of the cartoons to engage and maintain student interest and involvement, with significant intervention effects in Australia (Newton *et al*., 2020, 2022; Slade *et al*., 2020). However, as the applicability of the behaviour change principles underpinning *OurFutures* in the Bogotá context was not explored in the current study, the next phase of program adaptation could assess these principles’ relevance in a cross-cultural context and examine the contextual nuances influencing behaviour change outcomes in educational settings. Additionally, while this study focused on reviewing one lesson from the *OurFutures Alcohol Module*, which was sufficient for the purposes of the current study—intended as the first step in guiding the adaptation of the *OurFutures Alcohol Module* to the Bogotá context—future research should involve testing the entire module with young Colombians and teachers to gain a broader understanding of its effectiveness, cultural adaptability and potential impact in the Bogotá context. Despite certain limitations, the use of quantitative and qualitative data in this study aided in contextualizing and strengthening the findings for a better understanding of the cultural adaptation of the *OurFutures* program within the Bogotá context. Additionally, this study, through the collection of valuable feedback from Bogotá youth and teachers, serves as an essential step towards informing the adaptation of an effective eHealth school-based alcohol prevention program for the Bogotá context. Such a program is currently non-existent, yet critical, particularly considering the absence of standardized alcohol prevention education in Colombia. Moving forward, additional consultations with students and teachers will be conducted to further refine and enhance the program.

## CONCLUSION

This study marks the first step in informing the adaptation of an effective eHealth alcohol prevention program to the Bogotá context by gathering feedback from young Colombians and teachers. The implications of the study extend to the broader field of alcohol prevention in Colombian schools, with the identified challenges and recommendations providing valuable insights for developing culturally tailored preventive interventions. The findings provide evidence that the *OurFutures Alcohol Module* could be an acceptable and engaging alcohol prevention strategy in the Bogotá context, once appropriately adapted. The next steps involve incorporating participant feedback into the iterative adaptation of the *OurFutures* program, ensuring its relevance in the Bogotá context, and evaluating the feasibility of the program. Collaboration with place-based end-users, ongoing evaluation and a commitment to cultural sensitivity will be crucial in shaping a sustainable and impactful alcohol prevention initiative for young Colombians.

## Supplementary Material

daae152_suppl_Supplementary_Appendices

## Data Availability

The data underlying this article cannot be shared publicly for the privacy of individuals who participated in the study.
