# Supplementary material for: A mixed-methods study among adolescents and teachers in Bogotá, Colombia: adapting the OurFutures Alcohol Program
Source: Health Promot Int. 2024 Nov 15;39(6):daae152. doi: 10.1093/heapro/daae152 (PMC11565203; doi:10.1093/heapro/daae152)
Supplement: daae152_suppl_Supplementary_Appendices [file daae152_suppl_supplementary_appendices.docx]

Supplementary materials

Table of Contents

[OurFutures Alcohol Module lesson content summary 2](#_Toc162631666)

[Alcohol factsheet (English version of original provided for publication purposes) 3](#_Toc162631667)

[Focus group guide 5](#_Toc162631668)

[Interview guide 7](#_Toc162631669)

[Teacher online survey 9](#_Toc162631670)

[Post focus group evaluation survey 33](#_Toc162631671)

[Appendix table 2 37](#_Toc162631672)

[Appendix table 3 37](#_Toc162631673)

[In-depth description of qualitative findings from Part 1 on the attitudes and perceptions towards alcohol use held by young Colombian people and their teachers 38](#_Toc162631674)

[Student and teacher acceptability data from questionnaires 41](#_Toc162631675)

[In-depth description of qualitative results from Part 2 on the relatability and acceptability of OurFutures within the Bogotán context and areas for improvement 42](#_Toc162631676)

# OurFutures Alcohol Module lesson content summary

Appendix Table 1

| Lesson | Content |
| --- | --- |
| 1 | - Standard drinks. - The Australian guidelines to reduce health risks from drinking alcohol. - Alcohol, the law, and underage drinking. - Responsible drinking and how to stay safe. - Societal pressures and expectations to drink alcohol. |
| 2 | - Prevalence and patterns of alcohol use in young people. - Reasons why young people choose to or choose not to consume alcohol. - Positive alternatives to using alcohol, including alcohol-free social activities. - Myths about alcohol. |
| 3 | - Short- and long-term risks of drinking too much alcohol. - Potential risks and harms in common teenage drinking scenarios. - Ways to prevent harm in common teenage drinking scenarios. - Sources of help for young people, including seeking help from a school counsellor. |
| 4 | - The myths and facts about alcohol. - Alcohol advertising tactics and regulations. |
| 5 | - Alcohol refusal skills. - Ways to minimise alcohol consumption. - Decision-making about whether to consume alcohol. - Examining different views on the consumption of alcohol. - Keeping yourself and others safe when using alcohol. |
| 6 | - Recognising the signs of an alcohol-related medical emergency. - What to do and who to contact if there is an alcohol-related medical emergency. - First aid procedures when someone is unwell after drinking alcohol. |

# Alcohol factsheet (English version of original provided for publication purposes)


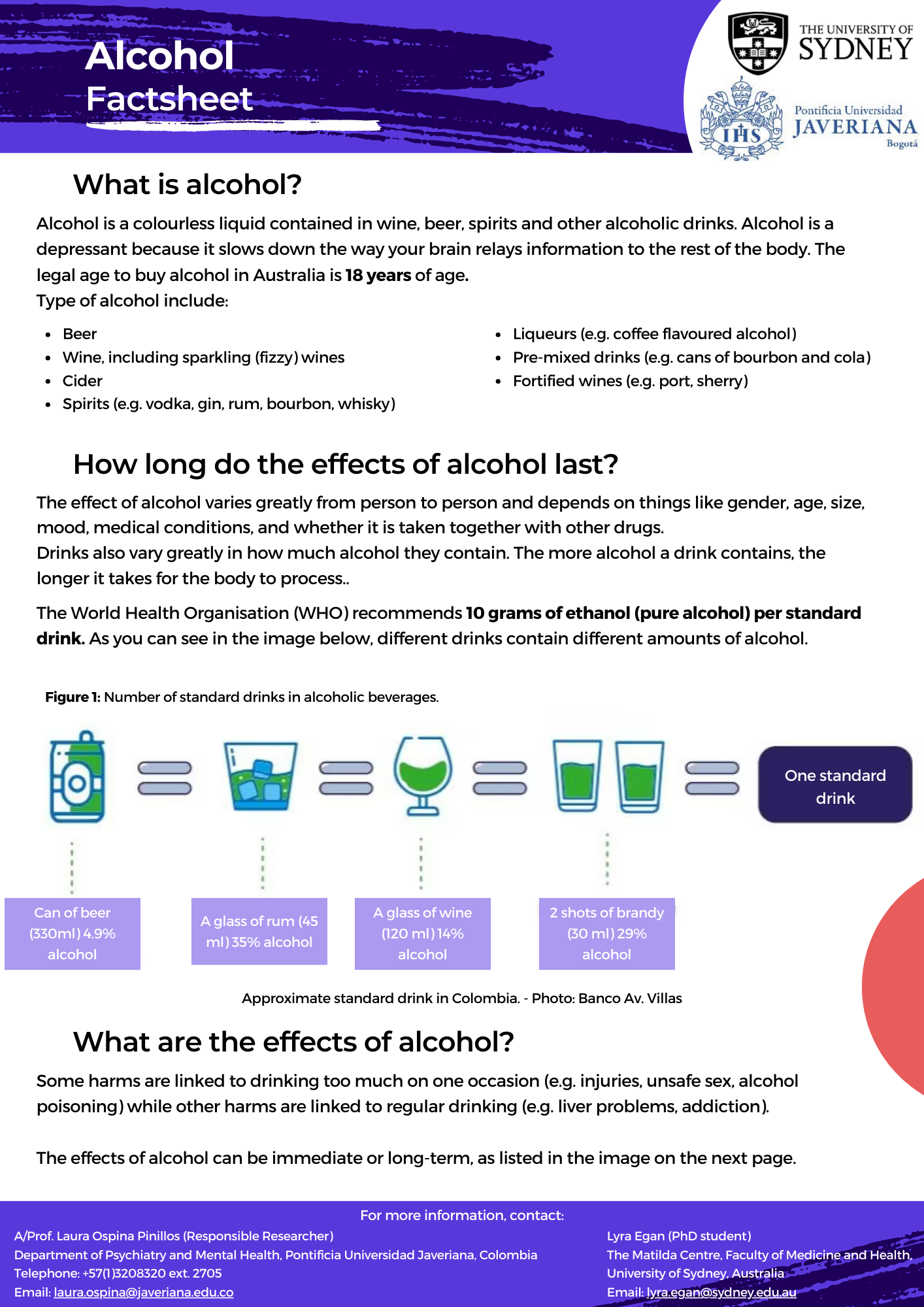


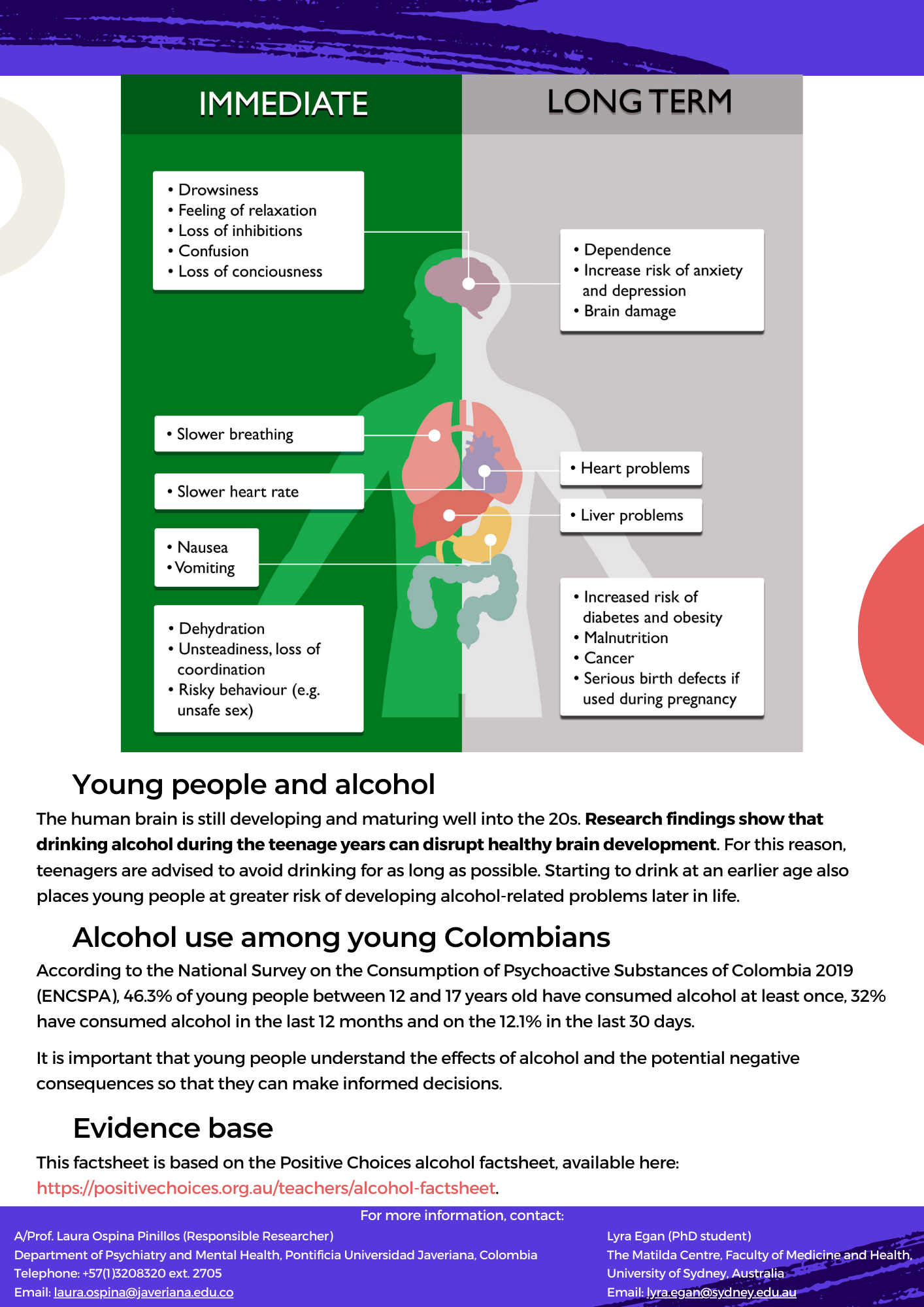


# Focus group guide

*Part 1: Exploring attitudes and perceptions about alcohol use (~20 minutes)*

1. What do you know about alcohol?

- Is there more alcohol in a unit of beer than in a unit of spirits?

1. Do you believe that young people (under the age of 18) can get into risky situations when consuming alcohol?

- Why/not

1. Do you believe that alcohol consumption is harmful to your body/health?

- Why/not

1. Do you think it is common for young people your age to consume alcohol?

- Would it be common for young people your age to engage in other health-risk behaviours such as tobacco smoking or e-cigarette use, eating nutrient-poor food (e.g. junk food/fried foods)?

1. What are some of the main reasons that someone your age might choose to consume alcohol?
2. What are some of the main reasons that someone your age might choose not to consume alcohol?
3. Where, or in what situations, do people your age most commonly consume alcohol?

- In these situations, would it be common to consume alcohol in addition to eating nutrient-poor food (e.g. junk food/fried foods) and/or smoke tobacco cigarettes or vape?

*Part 2: Going through one lesson from the OurFutures Alcohol Module (~25 minutes)*

- The researchers will display one of OurFutures Alcohol Module cartoon lessons on a screen and click through each slide (approximately 20 minutes).
- As the speech bubbles are translated into Spanish using the OurFutures Kanzi in-built translation software, students will be able to read the speech bubbles on each slide (approximately 100 slides in total).
- Following the cartoon, the researchers will display one of the interactive activities on a screen and prompt students to respond to the questions (approximately 5 minutes).

*Part 3: Relatability and acceptability of OurFutures, brainstorming new ideas for characters and storylines (~45 minutes)*

General feedback

1. Overall, how would you rate the OurFutures? (e.g. very good, average, very poor)

Storylines

1. How much did you like the storylines in the lessons? (e.g. liked a lot, disliked a little)
2. Were there any scenarios you found especially interesting or memorable?

- Why was this especially memorable?
- Would you change anything about the way this scenario was addressed in the cartoon?

1. How believable and realistic were the storylines for people your age? (e.g. completely believable and realistic, unsure, somewhat unbelievable and realistic)
2. What changes would you make to make the storylines more believable or realistic?
   - 1. E.g. the place where alcohol consumption would occur (e.g., at school, at someone’s house, at a party etc.)
     2. E.g. What other characters would be involved and what would their role be?
     3. How many of the friendship group would you expect to bring alcohol?
     4. Where do you think they would have gotten their alcohol from?
     5. What problems might the characters face?

If students can’t identify any, ask “what ifs” (e.g., What if someone didn’t want to consume alcohol but they were being peer pressured at a party?)

1. How might they overcome those problems?

Characters

1. How much did you like the characters in the lesson(s)? (e.g. liked a lot, disliked a little)
2. Which character do you relate to most and why?
3. Do you think other people in your age group would be able to relate to the characters? Why/why not?
   - 1. Are they representative of young people you know? How/why not?
        - Do they have different personality traits?
        - Do they look the same?
     2. Are they representative of who you think would consume / not consume alcohol? How/why not?
        - Do they display the same behaviours e.g. other health risk behaviours (e.g. tobacco smoking, e-cigarette use, nutrient-poor food consumption)? Do they experience negative effects from any of these behaviours and/or alcohol use?
        - Do they display the same health seeking behaviours e.g. staying physically active
        - When worried about their own or someone else’s alcohol consumption, do they seek out advice or help? How/why not?
        - What is their friendship group like? (e.g. do they have many friends? What are their friends into? Where do they hang out?)
        - What is their home life like? (e.g., Do they live with a parent/s? do they have siblings?)
     3. What are some other possible names for these characters?
4. Is there anything about the characters you would want to change?
5. Do you feel represented in the cartoons? If not, what could be included to improve the representation?

Language

1. Do you think the language used in the cartoon scripts was appropriate for young people your age?
2. Are there any specific words or phrases that you would like to see included in the cartoons?

# Interview guide

*Part 1: Exploring attitudes and perceptions about alcohol use and prevention (~15 minutes)*

1. What role does alcohol have in Colombian culture?
   1. Is it normalised? (for young people to consume alcohol with family?)
2. Do you believe it is common for Colombian youth to drink alcohol?
3. Does it differ by geographical location or socioeconomic status e.g. cities vs rural areas, low SES vs high SES?
4. Would initiation of alcohol use coincide with other health risk behaviours such as tobacco smoking or e-cigarette use, eating nutrient-poor food (e.g. junk food/fried foods)? Why/why not?
5. Do you know of evidence-based alcohol education / prevention resources available and accessible to Colombian youth?
   1. What are they? Who facilitates?
      1. Do you feel comfortable sharing these?
6. With regards to the current alcohol curriculum for Colombian youth, could you please describe what it involves and how it is taught?
   1. What topics are covered? (e.g. harm minimisation strategies)
   2. How much time is allocated to deliver alcohol curriculum?
   3. How is it delivered? (e.g. via prescribed texts in class, or internet resources/programs)
7. How likely are you to use an evidence-based digital alcohol prevention program specific to Colombian youth when teaching Colombian youth about alcohol?
   1. Do you foresee any barriers to using such a program?

*Part 2: Relatability and acceptability of OurFutures, brainstorming new ideas for characters and storylines (~45 minutes)*

1. **Which lesson(s) did you view?** (i.e. 1-6)
   - 1. Did you review any of the other resources e.g. teacher and student summaries, activities?
2. Overall, how would you rate the lessons? (i.e. very good, average, very poor)
3. What did you like or not like?
4. How believable and relevant do you think that the scenarios and examples within the cartoons would be for Colombian youth aged 11 to 13 years? (regarding lesson reviewed)
   - 1. Why/Why not?
     2. Ask about storylines and characters, anything to change, add (e.g. representation of different characters, was it believable and realistic)
5. Do you think the language used in the cartoon scripts was appropriate for Colombian youth aged 11 to 13 years?
6. Are there any specific words or phrases that you would like to see included in the cartoons?
7. Do you think the educational content is reasonable and appropriate for Colombian youth aged 11 to 13 years?
   - 1. Why/Why not?
8. Do you think that most Colombian youth aged 11 to 13 years would be able to understand and remember the concepts being taught?
   - 1. Why/Why not?
9. Do you think the length of each lesson (20 minutes plus an optional 20-minutes of activities) and the program (6 lessons) adequately covers the concepts being taught?
   - 1. Why/Why not?
10. Do you like the format in which this is delivered?
11. Would this be a program which you would deliver with your students?
    - 1. Why/Why not?
      2. E.g. what would make it more enticing?
12. Would you be interested in participating in a co-design workshop (e.g. design characters, review or change cartoon scripts) aimed at creating an adaptation to the OurFutures Alcohol Module?
    - 1. If so, would you also be interested in participating in a pilot trial of the adapted OurFutures Alcohol Module?
      2. If you would like to be contacted to participate in the co-design workshop and/or pilot trial please let us know your preferred contact details (i.e. address, email or phone number)

# Teacher online survey

**Part 1: Information About You**

| **Number of years’ experience teaching** |  |
| --- | --- |

**Part 2: Exploring attitudes and perceptions about alcohol use** 

1. What role does alcohol have in Colombian culture? Is it normalised? (for young people to consume alcohol with family etc?) *Please record as many reasons as you can think of.*

|  |
| --- |
|  |
|  |
|  |
|  |
|  |
|  |
|  |
|  |
|  |

1. Do you think it is common for Colombian youth to drink alcohol?

- Yes
- No

1. Would initiation of alcohol use coincide with other health risk behaviours such as tobacco smoking or e-cigarette use, eating nutrient-poor food (e.g. junk food/fried foods)? Why/why not?

- Yes
- No

Please explain response to this question (i.e. why/why not?)

|  |
| --- |
|  |
|  |

1. Is consumption of alcohol among Colombian youth similar across those living in different geographical locations i.e. cities vs rural areas?

- Not at all similar
- Somewhat similar
- Very similar
- Extremely similar

Please explain your response to this question

|  |
| --- |
|  |
|  |

1. Is consumption of alcohol among Colombian youth similar across those from different socioeconomic status groups e.g. low SES vs high SES?

- Not at all similar
- Somewhat similar
- Very similar
- Extremely similar

Please explain your response to this question

|  |
| --- |
|  |
|  |

1. Do you know of evidence-based alcohol education / prevention resources available and accessible to Colombian youth?

- Yes
  - - Please explain your response to this question (e.g. what are they and who facilitates them?)

|  |
| --- |
|  |
|  |

- No
- Unsure

1. With regards to the current alcohol curriculum for Colombian youth, could you please describe what it involves and how it is taught? *Please include information on the topics covered (e.g. harm minimisation strategies), time allocated to deliver the alcohol curriculum, and method of delivery (e.g. via prescribed texts in class, or internet resources/programs)*

|  |
| --- |
|  |
|  |
|  |
|  |
|  |

1. How likely are you to use an evidence-based digital alcohol education program specific to Colombian youth when teaching Colombian youth about alcohol?

- Not at all
- Slightly
- Moderately
- Very
- Extremely

Please explain your response to this question (e.g. barriers or motivators):

|  |
| --- |
|  |
|  |
|  |
|  |
|  |

**Part 3: The OurFutures Alcohol Module**

**After viewing the cartoon lessons please answer the following questions.**

1. **Please indicate which of the following you viewed (cartoons mandatory, others are optional):**

| Lesson 1 <if applicable>   - Online Cartoon - Teacher Summary - Student Summary - Activities | Lesson 2 <if applicable>   - Online Cartoon - Teacher Summary - Student Summary - Activities |
| --- | --- |
| Lesson 3 <if applicable>   - Online Cartoon - Teacher Summary - Student Summary - Activities | Lesson 4 <if applicable>   - Online Cartoon - Teacher Summary - Student Summary - Activities |
| Lesson 5 <if applicable>   - Online Cartoon - Teacher Summary - Student Summary - Activities | Lesson 6 <if applicable>   - Online Cartoon - Teacher Summary - Student Summary - Activities |

**FIRST IMPRESSIONS**

1. **Overall, how would you rate the lessons?**

- Very good
- Good
- Average
- Poor
- Very poor

1. **What are your first impressions of the module?**

|  |
| --- |
|  |
|  |
|  |
|  |
|  |

1. **What did you like?**

|  |
| --- |
|  |
|  |
|  |
|  |
|  |

1. **What didn’t you like?**

|  |
| --- |
|  |
|  |
|  |
|  |
|  |

**ACCEPTABILITY FOR STUDENTS**

1. **Do you think that the scenarios and examples within the cartoons would be believable and relevant for Colombian youth aged 11 to 13 years?**

| **Lesson 1** <if applicable>   - Yes - No     **Why/Why Not?** |
| --- |
|  |
|  |
|  |
|  |
|  |
|  |
|  |
|  |
|  |
|  |
|  |
|  |
|  |
|  |
| **Lesson 2** <if applicable>   - Yes - No   **Why/Why not?** |
|  |
|  |
|  |
|  |
|  |
|  |
|  |
|  |
|  |
|  |
|  |
|  |
|  |
|  |
|  |

| **Lesson 3** <if applicable>   - Yes - No     **Why/Why Not?** |
| --- |
|  |
|  |
|  |
|  |
|  |
|  |
|  |
|  |
|  |
|  |
|  |
|  |
|  |
|  |
| **Lesson 4** <if applicable>   - Yes - No   **Why/Why not?** |
|  |
|  |
|  |
|  |
|  |
|  |
|  |
|  |
|  |
|  |
|  |
|  |
|  |
|  |
|  |
| **Lesson 5** <if applicable>   - Yes - No   **Why/Why not?** |
|  |
|  |
|  |
|  |
|  |
|  |
|  |
|  |
|  |
|  |
|  |
|  |
|  |
|  |
|  |
| **Lesson 6** <if applicable>   - Yes - No   **Why/Why not?** |
|  |
|  |
|  |
|  |
|  |
|  |
|  |
|  |
|  |
|  |
|  |
|  |
|  |
|  |
|  |

**LANGUAGE**

1. **Do you think the language used in the cartoon scripts was appropriate for Colombian youth aged 11 to 13 years?** Please recommend any specific words or phases that should be included

| **Lesson 1** <if applicable>   - Yes - No     **Why/Why Not?** |
| --- |
|  |
|  |
|  |
|  |
|  |
|  |
|  |
|  |
|  |
|  |
|  |
|  |
|  |
|  |
| **Lesson 2** <if applicable>   - Yes - No   **Why/Why not?** |
|  |
|  |
|  |
|  |
|  |
|  |
|  |
|  |
|  |
|  |
|  |
|  |
|  |
|  |
|  |

| **Lesson 3** <if applicable>   - Yes - No     **Why/Why Not?** |
| --- |
|  |
|  |
|  |
|  |
|  |
|  |
|  |
|  |
|  |
|  |
|  |
|  |
|  |
|  |
| **Lesson 4** <if applicable>   - Yes - No   **Why/Why not?** |
|  |
|  |
|  |
|  |
|  |
|  |
|  |
|  |
|  |
|  |
|  |
|  |
|  |
|  |
|  |
| **Lesson 5** <if applicable>   - Yes - No   **Why/Why not?** |
|  |
|  |
|  |
|  |
|  |
|  |
|  |
|  |
|  |
|  |
|  |
|  |
|  |
|  |
|  |
| **Lesson 6** <if applicable>   - Yes - No   **Why/Why not?** |
|  |
|  |
|  |
|  |
|  |
|  |
|  |
|  |
|  |
|  |
|  |
|  |
|  |
|  |
|  |

**EDUCATIONAL CONTENT**

1. **Do you think the educational content is reasonable and appropriate for Colombian youth aged 11 to 13 years?**

| **Lesson 1** <if applicable>   - Yes - No     **Why / Why not?** |
| --- |
|  |
|  |
|  |
|  |
|  |
|  |
|  |
|  |
|  |
|  |
|  |
|  |
|  |
|  |
|  |
|  |
| **Lesson 2** <if applicable>   - Yes - No     **Why / Why not?** |
|  |
|  |
|  |
|  |
|  |
|  |
|  |
|  |
|  |
|  |
|  |
|  |
|  |

| **Lesson 3** <if applicable>   - Yes - No     **Why/Why Not?** |
| --- |
|  |
|  |
|  |
|  |
|  |
|  |
|  |
|  |
|  |
|  |
|  |
|  |
|  |
|  |
| **Lesson 4** <if applicable>   - Yes - No   **Why/Why not?** |
|  |
|  |
|  |
|  |
|  |
|  |
|  |
|  |
|  |
|  |
|  |
|  |
|  |
|  |
|  |
| **Lesson 5** <if applicable>   - Yes - No   **Why/Why not?** |
|  |
|  |
|  |
|  |
|  |
|  |
|  |
|  |
|  |
|  |
|  |
|  |
|  |
|  |
|  |
| **Lesson 6** <if applicable>   - Yes - No   **Why/Why not?** |
|  |
|  |
|  |
|  |
|  |
|  |
|  |
|  |
|  |
|  |
|  |
|  |

**LEARNING**

1. **Do you think that most Colombian youth aged 11 to 13 years would be able to understand and remember the concepts being taught?**

| **Lesson 1** <if applicable>   - Yes - No   **Why / Why not?** |
| --- |
|  |
|  |
|  |
|  |
|  |
|  |
|  |
|  |
|  |
|  |
|  |
|  |
|  |
|  |
| **Lesson 2** <if applicable>   - Yes - No     **Why / Why not?** |
|  |
|  |
|  |
|  |
|  |
|  |
|  |
|  |
|  |
|  |
|  |
|  |
|  |
|  |
|  |
|  |

| **Lesson 3** <if applicable>   - Yes - No     **Why/Why Not?** |
| --- |
|  |
|  |
|  |
|  |
|  |
|  |
|  |
|  |
|  |
|  |
|  |
|  |
|  |
|  |
| **Lesson 4** <if applicable>   - Yes - No   **Why/Why not?** |
|  |
|  |
|  |
|  |
|  |
|  |
|  |
|  |
|  |
|  |
|  |
|  |
|  |
|  |
|  |
| **Lesson 5** <if applicable>   - Yes - No   **Why/Why not?** |
|  |
|  |
|  |
|  |
|  |
|  |
|  |
|  |
|  |
|  |
|  |
|  |
|  |
|  |
|  |
| **Lesson 6** <if applicable>   - Yes - No   **Why/Why not?** |
|  |
|  |
|  |
|  |
|  |
|  |
|  |
|  |
|  |
|  |
|  |
|  |

**LENGTH**

1. **Do you think the length of each lesson (20 minutes plus an optional 20-minutes of activities) and the program (6 lessons) adequately covers the concepts being taught?**

| **Lesson 1** <if applicable>   - Yes - No     **Why / Why not?** |
| --- |
|  |
|  |
|  |
|  |
|  |
|  |
|  |
|  |
|  |
|  |
|  |
|  |
|  |
|  |
| **Lesson 2** <if applicable>   - Yes - No     **Why / Why not?** |
|  |
|  |
|  |
|  |
|  |
|  |
|  |
|  |
|  |
|  |
|  |
|  |
|  |
|  |
|  |
|  |
|  |

| **Lesson 3** <if applicable>   - Yes - No     **Why/Why Not?** |
| --- |
|  |
|  |
|  |
|  |
|  |
|  |
|  |
|  |
|  |
|  |
|  |
|  |
|  |
|  |
| **Lesson 4** <if applicable>   - Yes - No   **Why/Why not?** |
|  |
|  |
|  |
|  |
|  |
|  |
|  |
|  |
|  |
|  |
|  |
|  |
|  |
|  |
|  |
| **Lesson 5** <if applicable>   - Yes - No   **Why/Why not?** |
|  |
|  |
|  |
|  |
|  |
|  |
|  |
|  |
|  |
|  |
|  |
|  |
|  |
|  |
|  |
| **Lesson 6** <if applicable>   - Yes - No   **Why/Why not?** |
|  |
|  |
|  |
|  |
|  |
|  |
|  |
|  |
|  |
|  |
|  |
|  |

**FORMAT**

1. **Do you like the format in which the *OurFutures* program is delivered?**

- Strongly disagree
- Disagree
- Neither disagree or agree
- Agree
- Strongly agree

Please explain your response to this question

|  |
| --- |
|  |
|  |

**Intention to use**

1. **Would this be a program which you would deliver with your students?**

- Strongly disagree
- Disagree
- Neither disagree or agree
- Agree
- Strongly agree

Please explain your response to this question

|  |
| --- |
|  |
|  |

# Post focus group evaluation survey

**Part 1: Information About You**

| **What is your gender?** | Female | Male | Non-binary | Other |
| --- | --- | --- | --- | --- |

| **What sex were you assigned at birth?** (i.e. the one that was originally specified on your birth certificate) | Female | Male | Prefer not to answer |
| --- | --- | --- | --- |

| **What is your age?** |  |
| --- | --- |

***Below you will see questions asking for your feedback on the OurFutures cartoons. Your responses to these questions are optional and you can respond to as little or as many as you like.***

**Part 2: The OurFutures Cartoons**

***Thinking about the OurFutures cartoon lessons that you viewed today:***

1. **Overall, how would you rate the OurFutures lessons?**

Very Good  Good  Average  Poor  Very Poor

1. **How much did you like the storylines in the lessons?**

Liked a lot

Liked a little

Neither liked nor disliked

Disliked a little

Disliked a lot

**Please list any changes you would make to the storylines:**

|  |
| --- |
|  |
|  |
|  |
|  |
|  |
|  |

1. **How believable and realistic were the storylines for people your age?**

Completely believable and realistic

Somewhat believable and realistic

Unsure

Somewhat unbelievable and realistic

Completely unbelievable and realistic

**Please list any changes to make the storylines more believable or realistic** (e.g. the location where alcohol is consumed, the characters that would be involved and their relationship, the issues they may face and how they overcome such issues)**:**

|  |
| --- |
|  |
|  |
|  |
|  |
|  |
|  |
|  |

1. **How much did you like the characters in the lessons?**

Liked a lot

Liked a little

Neither liked nor disliked

Disliked a little

Disliked a lot

**Which character did you like the most? ___________________**

1. **How relatable were the characters in the lessons?**

Extremely relatable

Very relatable

Neutral

Very unrelatable

Not at all relatable

**Please list any changes you would make to the characters** (e.g. their personality traits, behaviours they engaged in such as eating healthy/unhealthy food, appearance, friendship group, names, more diversity of cultural backgrounds or different beliefs)**:**

|  |
| --- |
|  |
|  |
|  |
|  |
|  |
|  |
|  |
|  |
|  |
|  |
|  |
|  |
|  |
|  |

1. **Do you think the language used in the cartoon scripts was appropriate for young people your age?**

Extremely appropriate

Very appropriate

Neutral

Very inappropriate

Not at all appropriate

**Please list any changes you would make to the language used in the cartoons** (e.g. specific words or phrases that should be used)**:**

|  |
| --- |
|  |
|  |
|  |
|  |
|  |
|  |
|  |

|  | Strongly  Disagree | Disagree | Neither agree nor disagree | Agree | Strongly Agree |
| --- | --- | --- | --- | --- | --- |
| 1. **Do you think that other people your age will understand the information in the lessons?** |  |  |  |  |  |
| 1. **Do you think other people your age will like the characters?** |  |  |  |  |  |
| 1. **Do you think that other people your age will find the cartoons an engaging way to learn?** |  |  |  |  |  |

**11. Are there any other changes that you would like to see, even minor things?**

|  |
| --- |
|  |
|  |
|  |
|  |
|  |
|  |

# Appendix table 2

Table 2: Summary of focus group participant characteristics

| School grade (by focus group) | Age (years) | Number of participants | Gender (%) | | |
| --- | --- | --- | --- | --- | --- |
|  |  |  | Male | Female | Other |
| Grade 5 | 11-13 (M=11.78 years; SD=0.63) | 10 | 50 | 50 | 0 |
| Grade 6 | 11-14 (M=12.33 years; SD=1.28) | 6 | 67 | 33 | 0 |
| Grade 7 | 13-15 (M=13.70 years; SD=0.71) | 10 | 60 | 30 | 10 |
| **Total** |  | **26** | **59** | **38** | **3** |

# Appendix table 3

Table 3: Summary of teacher questionnaire and interview data on attitudes and perceptions regarding alcohol consumption among young people and education in Colombia.

| Question | Total  N (%) |
| --- | --- |
| Is it common for Colombian youth to drink alcohol? (% yes) | 10 (100%) |
| Would initiation of alcohol consumption coincide with other risk behaviours e.g. tobacco smoking, use e-cigarettes, eat junk food? (% yes) | 10 (100%) |
| Is alcohol consumption among Colombian youth similar across those living in different geographical locations i.e. cities vs rural areas? (% very/extremely similar) | 6/9 (67%) |
| Is alcohol consumption among Colombian youth similar across those from different socioeconomic status groups? (% very/extremely similar) | 4/10 (40%) |
| Do you know of evidence-based alcohol education / prevention resources available and accessible to Colombian youth? (% yes) | 1/10 (10%) |
| How likely are you to use an evidence-based digital alcohol education program specific to Colombian youth when teaching Colombian youth about alcohol? (% very/extremely likely) | 8/10 (80%) |

# In-depth description of qualitative findings from Part 1 on the attitudes and perceptions towards alcohol use held by young Colombian people and their teachers

i) Alcohol has cultural significance in Colombia.

According to both teachers and students, alcohol holds a prominent and symbolic role in Colombian culture. Celebratory occasions, from family gatherings to social events, often involve alcohol consumption. Focus group participants frequently shared that their first experience trying alcohol was during special events such as birthdays or Christmas. Teachers discussed the pervasive influence of alcohol in Colombian culture and its early exposure among its youth, and suggested that some parents view introducing alcohol early as promoting responsible consumption or as a cultural practice.

‘*Unfortunately, alcohol is part of Colombian culture, it’s seen in any kind of gathering, from beer to cocktails. It’s also very regrettable that it’s considered normal for children and teenagers to consume alcoholic beverages at family gatherings, with some saying it’s better for them to get used to it.*’ (Teacher 6)

Grade 7 participants generally perceived that parties among their age group always involved alcohol. This aligned with teachers’ comments on a societal expectation in Colombia that a celebration or gathering is incomplete without alcohol.

‘*A party without alcohol no, [doesn’t exist].*’ (Grade 7 focus group)

‘*…here in Colombia there is, perhaps, the custom or the culture that without alcohol one cannot have a good time at a party, in a meeting, in things like that. So, yes, generally the incidence of alcohol consumption in Colombia is very strong in any scenario*.’ (Teacher 2)

ii) Developmental stages and initiations

Teachers unanimously perceived alcohol consumption as common among Colombian youth, with initiation often occurring in family settings with minimal geographical variations, although the type of alcohol consumed may differ. In urban areas, young people have access to a variety of alcoholic beverages, while rural areas see a tradition of consuming local drinks like masato, chicha, or guarapo, which depending on the fermentation time, can have high alcohol content. Socioeconomic status, on the other hand, was generally perceived to shape both the onset age and type of alcohol consumed, with high-income families affording non-adulterated alcohol, while those from lower socioeconomic levels may resort to consuming adulterated alcohol or creating mixtures.

‘*Alcohol consumption among young Colombians normally first occurs in family spaces, it is the grandparents, uncles and cousins who accompany this first consumption, [rather than] differences in consumption by geographical locations,…what changes in the socioeconomic strata is the type of liquor consumed, well children from low socioeconomic strata consume more beer or low-priced liquor, while those in higher socioeconomic strata consume higher quality and priced liquor.*’ (Teacher 3)

*‘In rural areas, it is allowed by tradition. In the city, due to the greater supply and little parental control.*’ (Teacher 7)

‘*…Honestly, my students drink to relieve the pressure they face. Students from other schools with less academic demand may drink for different reasons from a younger age, and their behaviour is somewhat sanctioned by society…Most of the kids I work with come from high-income families that can afford at least non-adulterated alcohol. In lower economic levels, there may be cases of consuming adulterated alcohol or making mixtures.* (Teacher 1)

In focus groups, participants generally perceived alcohol consumption was common among their peers, particularly the grade 7 group. Grade 5 participants associated the initiation age with family influence and parental consent, ranging from 10 to 15 years. Grade 7 participants rejected initiation before 10, indicating inappropriateness of introducing alcohol at that age, suggesting an initiation age between 12 and 16. In contrast, grade 6 participants associated alcohol consumption with ages 16 and 17; however, contextual factors such as exposure to negative role models or neighbourhood circumstances influenced their perceptions.

‘*…over there sometimes [they start drinking] from the age of 14, and due to bad influence.*’(Grade 6 focus group)

‘*I live in [a neighbourhood where you] …even see small children like 8, 9 years old [drink alcohol]*. (Grade 6 focus group)

iii) Alcohol consumption influences and practices

Focus group participants perceived a range of motivations for alcohol consumption among their peers including experiencing feelings of depression, seeking independence, to be cool, to rebel, or satisfy curiosity. Social modelling, including adults consuming alcohol in spaces with young people was perceived to tempt young people to drink. Participants noted that young people often consume alcohol in settings where adults are drinking, and witnessing peers enjoy alcohol was perceived as a motivator for others to try it.

‘*Also because I know, you also see several young people, and it seems that the others inspire them to try that, and well, they seem to like it, I don’t know, and they keep trying it.*’ (Grade 6 focus group)

Furthermore, participants across all grades perceived the combination of alcohol with other substances as common, such as cocaine, cannabis, cigarettes, e-cigarettes, energy drinks, and junk food. Although grade 5 participants primarily discussed this phenomenon in the context of adults whereas grades 6 and 7 generalised it to people engaging in alcohol consumption.

‘*What I know is that also when a person drinks a lot of alcohol it causes them to try certain amounts of substances like “perico”, like cocaine.*’ (Grade 6 focus group)

Teachers also commented on the commonality of young people combining alcohol with other substances, particularly cigarettes and other psychoactive substances.

‘*…it's very, very evident that they consume alcohol along with cigarettes initially, or in addition to that, they consume some other type of psychoactive substance, either to enhance the effect of alcohol or to keep themselves a bit stabilised*.’ (Teacher 2)

iv) Health awareness and risk perception

Focus group participants universally perceived there are immediate risks when young people consume alcohol, such as violence and getting robbed. In grades 6 and 7, this perception extended to encompass long-term dangers, specifically cancer, traffic accidents, and death.

‘*There are some [people] who know how to handle alcohol, and others who don't, those who don't know how to handle alcohol, …go crazy, they pick fights, …those who don't know how to handle alcohol, it's just that they fall asleep on the street and that's why they get robbed*’ (Grade 6 focus group)

Interviewer: ‘*[You said] that it is harmful to health. What else?*

Participant: *[It] causes cancer.*

Interviewer: *it causes cancer, yes.*

Participant: *An addiction.’*

(Grade 7 focus group)

Despite these concerns expressed by focus group participants, some teachers perceived a very low-risk perception associated with alcohol use in Colombian society, especially among minors, highlighting family influence as a contributing factor.

‘*Simultaneously, in people's risk perception scales regarding how serious consuming alcohol is, it is inversely proportional. Among all drugs, alcohol has the lowest risk perception, around 10% or a little less. This high frequency of consumption, especially among minors, is often linked to family influence*.’ (Teacher 1)

# Student and teacher acceptability data from questionnaires

Appendix Table 4: Summary of student questionnaire data

| Question | n (%) |
| --- | --- |
| Overall rating of lesson (% good/very good) | 25/26 (96%) |
| How much did you like the storyline? (% liked a little/lot) | 25/26 (96%) |
| How believable and realistic was the storyline? (% completely/somewhat) | 22/25 (88%) |
| How much did you like the characters in the lesson? (% liked a little/lot) | 25/26 (96%) |
| How relatable were the characters in the lessons? (% very/extremely relatable) | 16/26 (62%) |
| Do you think the language used in the cartoon scripts was appropriate for young people your age? (% very/extremely appropriate) | 15/26 (58%) |
| Do you think that other people your age will understand the information in the lessons? (% agree/strongly agree) | 13/22 (59%) |
| Do you think other people your age will like the characters? (% agree/strongly agree) | 9/21 (43%) |
| Do you think that other people your age will find the cartoons an engaging way to learn? (% agree/strongly agree) | 13/22 (59%) |

Appendix Table 5: Summary of teacher questionnaire and interview data on the relatability and acceptability of the *OurFutures Alcohol Module* within the Bogotán context.

| Question | Total (n=10),  n (%) |
| --- | --- |
| Overall rating of lesson (% good/very good) | 8/9 (89%) |
| Do you think that the scenarios and examples within the cartoons would be believable and relevant for Colombian youth aged 11 to 13 years? (% yes) | 7/10 (70%) |
| Do you think the language used in the cartoon scripts was appropriate for Colombian youth aged 11 to 13 years? (% yes) | 6/10 (60%) |
| Do you think the educational content is reasonable and appropriate for Colombian youth aged 11 to 13 years? (% yes) | 9/10 (90%) |
| Do you think that most Colombian youth aged 11 to 13 years would be able to understand and remember the concepts being taught? (% yes) | 4/7 (57%) |
| Do you think the length of each lesson (20-minutes plus optional 20-minutes of activities) and the program (6 lessons) adequately covers the concepts being taught? (% yes) | 7/10 (70%) |
| Do you like the format in which the OurFutures program is delivered? (% agree/strongly agree) | 5/7 (71%) |
| Would this be a program which you would deliver with your students? (% agree/strongly agree) | 8/10 (80%) |

# In-depth description of qualitative results from Part 2 on the relatability and acceptability of OurFutures within the Bogotán context and areas for improvement

ii) The language needs to be contextualised

Both teachers and focus group participants emphasised the importance of using familiar, colloquial vocabulary and culturally relevant language in OurFutures. While some teachers perceived the existing language as relatable, others pointed out that the current language was at times too technical or refined and suggested incorporating jargon and changing alcohol-related terms to align with everyday language used by young Colombians.

‘*It is necessary to recognise and use vocabulary that is familiar to young Colombians.*’ (Teacher 3)

‘*Yes, for example, here in Colombia, we say "trago," "chorro," "aguardiente," nothing too refined or delicate when talking about alcohol…and I see that in the cartoons, there was a language that was either too technical or too refined, too delicate*.’ (Teacher 2)

‘*It would be very important to have the Colombian accent, the physical appearance of the characters, and their names, also the fashion*…’ (Grade 7 focus group)

Focus group participants recommended culturally relatable alternatives to replace original character names, including “Sebastián” for Mike, “Valentina” for Jane, “Tomás” for Tom, “Sara” for Claire, and “David” for Dave. Moreover, they recommended integrating colloquial or Bogotán expressions to substitute words that either did not translate well from English to Spanish or reflected vocabulary and expressions from Spain, rather than those commonly used in Colombia. For instance, they identified the term “guay” (“cool” in English) as characteristic of Spanish from Spain, suggesting more typical Colombian expressions to replace it, including “chévere”, “chimba”, or “bacano”. Participants also recommended using slang words, such as “farra” for a party, referring to the police as “los tombos” (similar to saying “the cops” in English), or saying “los aguacates,” which literally means “the avocadoes” in English but is associated with the olive-green police uniform. Additionally they proposed replacing terms like “Criminales” to refer to criminals with “Malandros”, and terms like “sexy” to describe physical attractiveness with “rica” (“nice” in English), “bonita” (“pretty” in English) or “hermosa” (“beautiful” in English).

iii) Aligning alcohol representation in OurFutures with Bogotán context

Focus group participants recommended adjusting the portrayal of alcoholic beverages in OurFutures to better resonate with Colombian culture. Suggestions included substituting instances of characters drinking vodka with locally familiar drinks such as “Aguardiente” (brandy in English), high-alcohol premixed drinks, and common beer brands. In a scene where characters mix vodka with orange juice, participants suggested replacing it with mixing “Aguardiente” with Gatorade. However, only grade 5 students discussed a preference for mixing beer with sweets, a trend they had seen on TikTok.

‘*There wouldn't be vodka, rather aguardiente*…’ (Grade 7 focus group)

Interviewer: ‘*But, then wait, if this was Aguardiente or brandy, would you mix it with orange juice or what would it be mixed with?*

Participant: *Here it is mixed with Gatorade*

Participant: *Gatorade*

…

Interviewer: *Anything else? To mix the brandy with*

Participant: *With sweets, yes*

Interviewer: *How so? With what sweet?*

Participant: *With jellybeans or like that?*

Interviewer: *So, one takes a jellybean and puts it in the liquor and drinks it?*

Participant: *I have seen it through TikTok videos*

(Grade 5 focus group)

iv) Narrative elements: cultural alignment and diversity (characters and stories)

Both focus group participants and teachers highlighted discrepancies in character representation and story elements, expressing concerns about their lack of alignment with the Bogotán context. Despite an appreciation for Mike’s character (followed by Claire, Jane, and Tom), focus group participants desired more characters and criticised the perceived affluence of existing ones. Recommendations included changes to clothing, hairstyles, and a broader diversity in appearances to better represent people their age.

‘*They were very posh, I would change that.*’ (Grade 5 focus group)

Teachers echoed the need for more realistic and diverse characters, expressing concerns about beauty stereotypes and relationship portrayals.

‘*…some characters that are not very evident in Colombian reality were included.*’ (Teacher 2)

‘*There were some little things that bothered me, I feel that there are some beauty stereotypes…the cute girl is the one they fall in love with,…so as not to continue reproducing these ideas they have that the only one with a boyfriend in the group is the slim cute girl*.’ (Teacher 1)

Both students and teachers suggested modifications to scenes, advocating for a shift from beach settings (more aligned with Australian contexts) to park environments resonating with young Colombians, particularly those in Bogotá. Teachers proposed including locations such as family gatherings to better reflect social dynamics in Colombia, and suggested framing scenes to portray families as unaware of their children consuming alcohol. While some scenarios were appreciated by teachers, with one stating they appreciated OurFutures’ effort in presenting realistic situations, other scenes were not as well received with one teacher raising concerns about the portrayal of absent parents.

‘…*They generally [drink] in parks. So, one scenario could be in parks, …I was also thinking maybe a family gathering…their behaviour is different when they are with family…compared to when they are completely alone, with no one's knowledge. So, I would believe that these scenarios can be framed in such a way*.’ (Teacher 2)

‘…*at no time did the [quiz questions] expect for you to write "don't drink" or "not to have gone to the party," but rather, perhaps when my friend said he was leaving [the party], I would go with him…So, I liked that, I thought it was very valuable that the situations they presented were realistic, that seemed very, very, very valid to me.*’ (Teacher 1)

‘*…it somewhat contradicts what parents might expect. I think, while the situation, for example, about girls drinking, one's mother being absent, the context of absent parents—parents are absent, but not absent like that. I mean, I don't see any of my students identifying with their mom being a flight attendant and being away.*’ (Teacher 1)

Similar to teachers, focus group participants found it unrealistic for a parent to be an air hostess, let alone go to New Zealand for a night, given the considerable distance from Colombia. They proposed scenarios where characters would have met each other in the neighbourhood, at school, at soccer, or at a party instead of at the beach. Additionally, grade 7 students stated that going to a park to continue the party after drinking would be unrealistic; instead, they proposed returning to someone’s house. Specific critiques were directed towards the scene involving intoxicated characters riding a shopping trolley, with participants unanimously perceiving this as unrealistic and suggesting a more plausible alternative, such as riding bikes.

Interviewer: ‘*So, would you use the shopping cart? Or do you think you would ride on something else, like a bicycle or something?*

Participant: *On a bicycle.*

Participant: *No, because we don't fit three, four on it.*

Participant: *One on the handlebars, one sitting on the saddle, and another on the back, and we go like that, and in the meantime, you can brake.*

Participant: *Easy.*

Participant: *Or two on the pedals and they drive.*

Participant: *Yes.*’

(Grade 6 focus group)

The importance of relatability in disciplinary actions was also emphasised, with participants suggesting that having parents take away phones would be a more realistic consequence if they were grounded or in trouble.

‘*No, she would take away my phone, the most valuable thing of a teenager right now*.’ (Grade 5 focus group)

v) Educational content

Regarding educational content, teachers generally appreciated the clarity and appropriateness of the content, and the framing of various authority figures beyond the police, such as parents, guardians, teachers, counsellors, and coordinators. This broad representation was perceived as an effective strategy to raise awareness about decision-making and social influence. However, one teacher perceived religious or spiritual elements were lacking from the content, and another expressed concerns about the advanced content and situations, deeming them inappropriate for certain age groups.

‘*Yes, the educational content, because it's a matter of raising awareness about decision-making…I found it interesting that they didn't only portray the police as an authority figure…authority figures are also at home, parents, guardians, here at school, teachers, counsellors, coordinators…I consider that part of the pedagogical sense is very well framed*.’ (Teacher 2)

‘*It is very advanced for the age of the children.*’ (Teacher 9)

‘*…for my students, I'm also a homeroom teacher for seventh grade, ranging from 13 to 14 years old; it wouldn't work at all. The content is a bit advanced, and in terms of the situation, showing them that might open windows they're not ready for yet. They wouldn't understand the situations very well.*’ (Teacher 1)

vi) Teachers appreciate the flexibility in delivery but call for improvements

Teachers expressed diverse perspectives on the format of OurFutures. Some valued the 20-minute cartoons as the core component and appreciated the flexibility in completing optional activities. They perceived this format as suitable for class schedules, especially when time constraints are a concern. Conversely, some called for additional activities, whereas others found the format lengthy and wanted more dynamic and engaging elements. The two teachers who reviewed OurFutures via the website said that the platform was slow.

‘*…it was cool that the central part was 20 minutes, I think that's what you can take advantage of in a class. In my classes, they are 50 minutes long, and in the end, between discipline, behaviour issues, what ends up being used in a class is about 20 minutes,…Perhaps doing more activities is a bit ambitious…I would think that it can be an alternative, but not everything will be achieved.*’ (Teacher 1)

Other recommendations from teachers included incorporating follow-up sessions, continuing evaluation for enhanced learning outcomes, and providing ongoing support and feedback.

‘*Follow-up and not abandoning the program.*’ (Teacher 4)

‘*Feedback and ongoing evaluation*.’ (Teacher 5)
